# Comparative analysis of NF-Y transcription factors in four orchids with expression insights from *Gastrodia elata*

**DOI:** 10.7717/peerj.21105

**Published:** 2026-03-20

**Authors:** Ailaiti Tuerxun, Xuewei Zhao, Xin He, Siren Lan, Zhongjian Liu

**Affiliations:** 1 College of Forestry, Fujian Agriculture and Forestry University, Fuzhou, China; 2 China Strait Horticulture Technology Innovation Hub, College of Landscape Architecture and Art, Fujian Agriculture and Forestry University, Fuzhou, China

**Keywords:** NF-Y gene family, Orchids, Floral organ development, Expression analysis

## Abstract

The NUCLEAR FACTOR Y (NF-Y) transcription factor family plays a crucial regulatory role in various aspects of plant development, physiological responses, and light signaling pathways. Nevertheless, there are limited reports on the characteristics and functions of NF-Ys in orchids. This study identified the NF-Y gene family in four orchids (*Gastrodia elata*, *Gastrodia menghaiensis*, *Platanthera guangdongensis*, and *Platanthera zijinensis*) and analyzed their structural domains, physicochemical properties, phylogenetic relationships, gene collinearity, and the *cis*-elements in their promoter region. A total of 91 NF-Ys were identified from four orchids, among which NF-Y gene numbers varied among orchid species, with relatively fewer NF-Y genes identified in fully mycoheterotrophic orchids compared with partially mycoheterotrophic taxa. Phylogenetic analysis classified these genes into three subfamilies, and the protein domains and gene structures of the same branch exhibited high similarity. The promoters of these NF-Ys are enriched with photoresponsive *cis*-elements. During floral organ development in *G. elata*, GelNFY2, GelNFY6, and GelNFY14 were significantly downregulated at the large bud stage (S2) but significantly upregulated in the lip of flowering stage (S4), suggesting their potential involvement in lip development. In conclusion, this study provides a valuable resource for further investigation into the regulatory functions of the NF-Y gene family in orchids.

## Introduction

**Figure 1:**
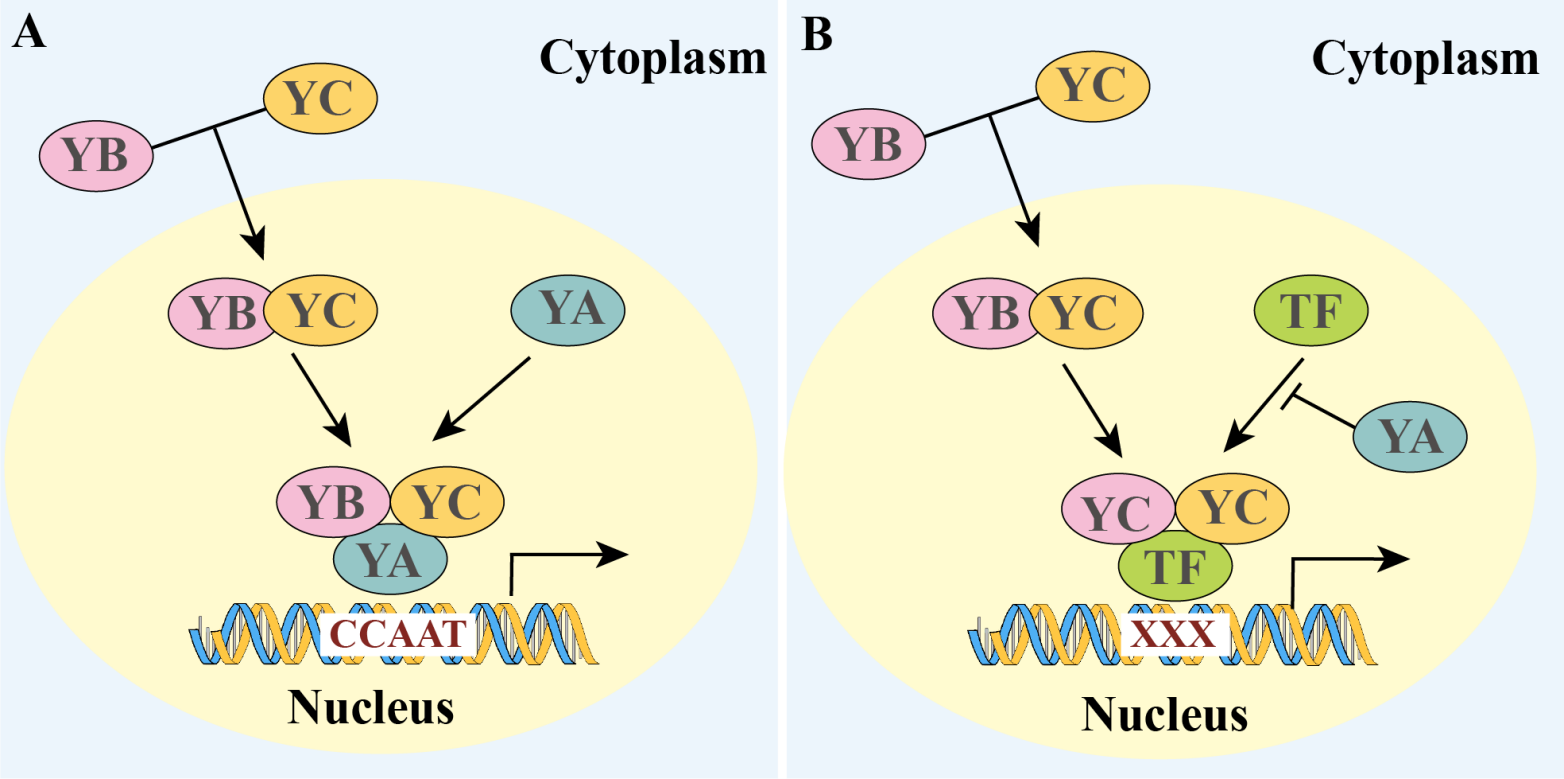
Regulatory mechanism of three NF-Y subfamilies. This figure was redrawn and modified based on Wu, Hou & Zhang (2025). All components were created using Adobe Illustrator.

Transcription factors (TFs) serve as key nodes in the gene regulatory network that can modulate specific pathways and morphogenesis in plants by dynamically adjusting spatiotemporal gene expression in response to environmental fluctuations (Koyama, 2023). The NUCLEAR FACTORY (NF-Y) transcription factor family plays a pivotal role in regulating plant development and physiology (Myers & Holt3rd, 2018). NF-Y transcription factors function as heterotrimeric complexes, recognizing and binding to the CCAAT consensus sequence. They are conventionally categorized into three subfamilies: NF-YA, NF-YB, and NF-YC (Laloum et al., 2014; Zanetti, Rípodas & Niebel, 2017). This classification suggests that genes within the same branch may share similar expression patterns and regulatory functions. Among these subfamilies, the NF-YB and NF-YC subunits contain histone fold domains (HFDs), which enable DNA interaction and facilitate nuclear localization (Nardini et al., 2013). Subsequently, NF-YA joins the complex to form a complete NF-Y complex, which can bind to DNA and regulate transcription (Myers & Holt 3rd, 2018; Nardini et al., 2013) (Fig. 1).

NF-Y is thought to play a role in processes such as flowering time, early seedling development, stress response, and hormone signaling (Jiang et al., 2023a; Jiang et al., 2023b; Li et al., 2022a; Li et al., 2023). The function of NF-Y has been extensively documented in various plants. Specifically, the NF-YA subfamily influences the sensitivity of *Arabidopsis* seeds to abscisic acid (ABA) during germination and regulates lateral root formation and development (Kumimoto et al., 2010; Siriwardana et al., 2014; Sorin et al., 2014). It has been demonstrated that different combinations of mutations and overexpression of NF-YB and NF-YC genes significantly altered the morphological and molecular phenotypes associated with ABA signaling (Kumimoto et al., 2013), and NF-YB6/9 has been shown to regulate vegetative cells and embryonic development of *Arabidopsis* (Ballif et al., 2011; Kwong et al., 2003). The overexpression of NF-YC subfamily member NF-YC10 in rice can enhance heat resistance, while NF-YC3/4/9 affects the establishment of plant photomorphogenesis (Myers et al., 2016). Extensive studies have highlighted the significant role that the NF-YB and NF-YC subunits play in regulating flowering time (Swain et al., 2017; Wenkel et al., 2006).

As one of the largest families of flowering plants in the world, orchids exhibit diverse flower morphologies and lifestyles, unique xerophytic physiologies, and complex mycorrhizal relationships (Li et al., 2022b; Yuan et al., 2018; Zhang et al., 2022). These characteristics not only make orchids crucial for ecosystem stability but also valuable in horticulture, pharmaceuticals, and traditional medicine. The continuous release of orchid genome data provides opportunities to explore the roles of various transcription factors in orchids. However, studies on floral development in saprophytic-like orchids remain limited. Although transcriptomic analyses, such as the study on transcriptomic heterochrony and cleistogamous flower development in *Gastrodia*, have provided important insights into floral developmental dynamics, gene family–level regulatory mechanisms underlying floral development are still poorly understood (Suetsugu et al., 2023). NF-Y transcription factors play important roles in diverse developmental processes in plants and have also been implicated in stress responses and flowering regulation in orchids, potentially through *cis*-elements in their promoter regions and interactions with key flowering-related genes (Jiang et al., 2023a). Nevertheless, the regulatory effects of NF-Ys in orchids was only reported in *Phalaenopsis* (Jiang et al., 2023a) and *Cymbidium sinense* (Wang et al., 2024). These results illustrate the pivotal role that NF-Ys play in stress resistance and floral development in orchids.

Fully mycoheterotrophic orchids exhibit pronounced morphological and physiological specialization, prompting the question of whether developmental regulatory gene families have experienced lineage-specific contraction or differential retention. Accordingly, we proposed the following testable expectations: (i) The number of members in the NF-Y gene family is reduced in fully mycoheterotrophic orchids relative to partially mycoheterotrophic species; (ii) despite an overall reduction in gene number, retained NF-Y genes exhibit conserved phylogenetic placement and syntenic relationships across orchid lineages; and (iii) NF-Y genes display subfamily-biased expression patterns across floral organs during key stages of flower development in *Gastrodia elata*. Thus, this study comprehensively analyzed the NF-Y gene family of four orchids (*Gastrodia elata*, *Gastrodia menghaiensis*, *Platanthera guangdongensis*, and *Platanthera zijinensis*). These findings contribute to a deeper understanding of the molecular mechanisms underlying floral organ development in orchids and provide valuable insights into the regulatory networks controlling flowering.

## Materials & Methods

### Plant materials and data sources

The plant materials of *G. elata* used in this study were wild-type plants grown in the greenhouse of the Forest Orchid Garden at Fujian Agriculture and Forestry University (temperature, 20–25 °C; photoperiod, 12 h light/12 h dark; illumination intensity, 200 lux). We collected the *G. elata* flowers perianth tube (PE), gynostemium/ovary (GY), and lip (LIP), at four floral organ developmental stages: the small bud stage (S1), the large bud stage (S2), initial bloom stage (S3), and the blooming stage (S4). For each tissue at each developmental stage, three independent biological replicates were collected from different plants. Subsequently, an immediate flash-freezing protocol employing liquid nitrogen was applied to these samples, followed by storage at −80 °C.

The original data related to *G. elata* was acquired from the National Center for Biotechnology Information (NCBI, https://www.ncbi.nlm.nih.gov/) (Yuan et al., 2018). The original data related to *G. menghaiensis* was acquired from the Figshare database (https://figshare.com/s/f759784e78e86ba71c7c) (Jiang et al., 2022). Genome sequences and whole genome assemblies for *P. guangdongensis* and *P. zijinensis* were downloaded from the NCBI database under BioProject PRJNA739531 (Li et al., 2022b). The raw data of transcriptome related to floral organ development in *G. elata* have been deposited in the China National GeneBank (CNGB) and are available at https://db.cngb.org/data_resources/project/CNP0008391 (DOI: 10.26036/CNP0008391). Additionally, the protein sequence files associated with NF-Y in *A. thaliana* and *Oryza sativa* were obtained from the Plant Transcription Factor Database (PlantTFDB, http://planttfdb.gao-lab.org/index.php?sp=Ath). The protein sequences of all identified genes are provided in Table S1.

### Identification of NF-Ys in orchid genomes

Using *A. thaliana* NF-Y proteins as reference sequences, we conducted a local BLAST search (*E*-value = 1  × 10^−5^) on the protein files of *P. guangdongensis*, *P. zijinensis*, *G. elata*, and *G. menghaiensis* genomes. The identified candidate NF-Ys in four orchids were further analyzed using the sequence alignment function of ESPript 3.0 (https://espript.ibcp.fr/ESPript/cgi-bin/ESPript.cgi) to screen for NF-Ys in *G. elata*. Sequences that did not contain NF-Y conserved domains or contained only incomplete forms of these domains were excluded (Chen et al., 2023). The remaining candidate sequences were then re-analyzed using BLASTP (https://blast.ncbi.nlm.nih.gov/Blast.cgi?PROGRAM=blastp&PAGE_TYPE=BlastSearch&LINK_LOC=blasthome) in NCBI to further confirm whether the obtained sequences belonged to the NF-Y gene family.

### Multiple sequence alignment and phylogenetic analysis

Multiple sequence alignment of NF-Y protein sequences was performed using MAFFT v7 with the automatic strategy selection (–auto) (Katoh & Standley, 2013), and the resulting alignment was subsequently trimmed using trimAl to remove poorly aligned regions with a gap threshold of 0.7 (Capella-Gutiérrez, Silla-Martínez & Gabaldón, 2009). The NF-Y protein sequences of *P. guangdongensis*, *P. zijinensis*, *G. elata*, *G. menghaiensis*, *A. thaliana*, and *O. sativa* were inferred using the maximum-likelihood (ML) method implemented in IQ-TREE v2 (Minh et al., 2020). The best-fit substitution model was selected automatically by ModelFinder, and branch support was assessed using 1,000 ultrafast bootstrap replicates (UFBoot) together with 1,000 SH-aLRT tests. The phylogenetic tree was visualized using the online tool Evolview (https://bio.tools/evolview) (Stephenson et al., 2010).

### NF-Y proteins physicochemical properties and gene structure analysis

The ProtParam tool from ExPASy (https://web.expasy.org/protparam/) was utilized to obtain the physicochemical properties of orchid NF-Y proteins. MEME (https://meme-suite.org/meme/tools/meme) was employed to analyze conserved motifs. Additionally, information regarding the number of exons, introns, coding sequences (CDSs), and untranslated regions (UTRs) for the NF-Y genes was acquired from the genome annotation file, and these data are visualized using the Gene Location Visualize (GTF/GFF) in TBtools (version 1.120).

### Collinearity and chromosomal location analysis of NF-Ys

To investigate the collinear associations between these species and identify NF-Y gene collinear blocks, we employed the One-Step MCScanX command in TBtools (version 1.120). Using the genome and gene annotation files of *P. guangdongensis*, *P. zijinensis*, *G. elata*, and *G. menghaiensis*, TBtools (version 1.120) was employed to extract and visualize the chromosomal locations of NF-Y genes (Chen et al., 2023).

### *Cis*-elements prediction for *NF-Y*s

The 2,000 bp upstream sequences of the orchid *NF-Y*s was extracted using TBtools (version 1.120). Potential *cis*-elements within the promoter regions of these *NF-Y*s were pinpointed using PlantCARE (http://bioinformatics.psb.ugent.be/webtools/plantcare/html/). Subsequent data analysis and visualization were performed using Excel 2021 and GraphPad Prism 9.0.

### Gene Ontology (GO) analysis

Functional annotation of genes was conducted using eggNOG-mapper (http://eggnog-mapper.embl.de/) based on the eggNOG 5.0 database (Cantalapiedra et al., 2021; Huerta-Cepas et al., 2019). Orthologous relationships were identified through sequence alignment, with bit-score and *E*-value thresholds applied to ensure annotation accuracy. Gene Ontology (GO) terms were assigned to proteins to classify them into relevant biological processes, molecular functions, and cellular components. The results of the GO annotation are provided in Table S4.

### Expression patterns and quantitative reverse transcription polymerase chain reaction analysis

To investigate the expression patterns of GelNFYs during *G. elata* floral development, RNA-Seq *via* Expectation Maximization (RSEM) was employed to quantify transcription levels (Li & Dewey, 2011). This involved computing the fragments per kilobase of transcript per million mapped reads (FPKM) and creating a comprehensive RNA-sequencing transcriptome database for various stages of flower parts. For RNA-seq analysis, three biological replicates were used for each treatment group to ensure reproducibility and statistical reliability. Statistical power analysis of the RNA-seq experimental design was conducted in R using the RNASeqPower package. With parameters set to an average read depth of 20 reads per gene, a coefficient of variation (CV) of 0.4, a fold change of 2 (log_2_FC = 1), and a significance level of α = 0.05, the analysis confirmed that the experimental design provided sufficient statistical power for reliable differential expression detection. Subsequently, TBtools (version 1.120) was used to generate a heatmap based on FPKM data.

The Quantitative reverse transcription polymerase chain reaction (qRT-PCr) analysis was conducted to delve deeper into the expression pattern of GelNFYs. Floral components of *G. elata* were collected at four distinct flowering stages, and total RNA extraction was performed using the Fast Pure Plant Total RNA Isolation Kit (Vazyme Biotech Co., Ltd., Nanjing, China). First-strand cDNA was synthesized using TransScript^®^ All-in-One First-Strand cDNA Synthesis SuperMix for quantitative PCR (TransGen Biotech, Beijing, China). Primers for candidate genes and internal reference genes for qRT-PCR were designed using Primer Premier 5. The specificity of these primers was subsequently validated through Primer-BLAST analysis conducted on NCBI (https://www.ncbi.nlm.nih.gov/tools/primer-blast/index.cgi?LINK_LOC=BlastHome). The qRT-PCR was performed using gene-specific primers on CFX96 Real-Time PCR (Bio-Rad, USA) with the ChamQ Blue Universal SYBR qPCR Master Mix (Nanjing Vazyme Biotech Co., Ltd., Nanjing, China), using the thermal cycling conditions of 95 °C for 30s followed by 40 cycles of 95 °C for 10s and 60 °C for 30s, and the GelActin (Gel003815) gene was used as an endogenous control. Each sample was set up with three biological replicates and three technical replicates. Details of the experimental design and validation parameters following the MIQE guidelines are provided in Table S8 (MIQE Checklist).

## Results

### Identification and phylogenetic analysis of the NF-Ys

**Figure 2:**
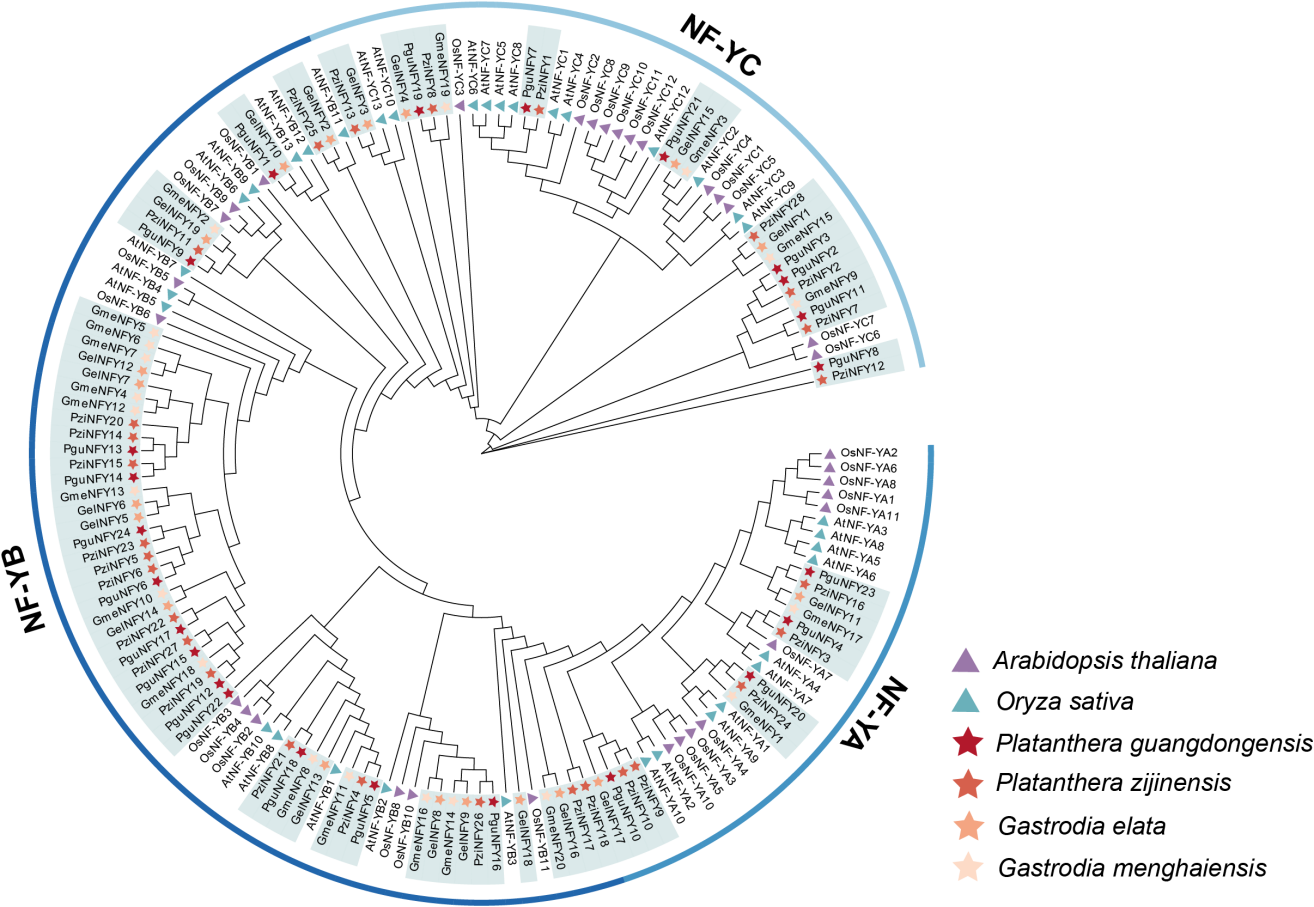
The NF-Y phylogenetic tree of six plants. The corresponding NF-Y protein sequences can be observed in Table S1.

A total of 91 NF-Y proteins (NF-Ys) were identified from four orchid genomes (Fig. 2), with 24 proteins in *P. guangdongensis* (PguNFYs), 28 proteins in *P. zijinensis* (PziNFYs), 19 proteins in *G. elata* (GelNFYs), and 20 proteins in *G. menghaiensis* (GmeNFYs). The NF-Ys were named according to their sequential arrangement on the chromosomes, from top to bottom (Table S2). To investigate the evolutionary relationships of NF-Y proteins, a ML phylogenetic tree was constructed using NF-Y protein sequences of 35 AtNFY proteins, 33 OsNFY proteins, and 91 orchid NF-Ys. Based on phylogenetic clustering and the established classification of the *A. thaliana* NF-Y gene family, the orchid NF-Y genes were divided into three subfamilies: NF-YA (13 orchid NF-Ys), NF-YB (56 orchid NF-Ys), and NF-YC (22 orchid NF-Ys). Specifically, the NF-YB subfamily comprised 13 GelNFYs, 14 GmeNFYs, 13 PguNFYs, and 16 PziNFYs, making it the most abundant NF-Y subfamily relative to NF-YA and NF-YC in the four orchid species analyzed. This expansion may be partially attributable to tandem duplication events, as exemplified by gene pairs (such as GmeNFY6 and GmeNFY7, PziNFY17 and PziNFY18). In contrast, the NF-YA and NF-YC subfamilies exhibited relatively fewer members, suggesting stronger evolutionary constraints on these two subfamilies. Furthermore, *P. guangdongensis* and *P. zijinensis* harbored more NF-YC genes than the two *Gastrodia* species, implying that gene loss in *Gastrodia* may have preferentially occurred within the NF-YA and NF-YC subfamilies.

### Analysis of NF-Y proteins physicochemical property

We analyzed the physicochemical properties of NF-Y proteins to gain further insights into their physical characteristics (Table S2). The analysis revealed that the amino acid length ranged from 96 aa (PguNFY22) to 465 aa (GmeNFY18), the molecular weight (Mw) ranged from 10.55 kDa (PguNFY22) to 51.07 kDa (GmeNFY18). The isoelectric point (pI) ranged from 4.60 (GelNFY10) to 10.72 (GmeNFY4), among which 52 NF-Y proteins were basic proteins. The instability index (II) ranged from 10.15 (PziNFY15) to 81.37 (PguNFY22), and aliphatic indexes (AI) ranged from 45.98 (PguNFY16) to 100.46 (GelNFY4). Furthermore, the physicochemical analysis indicated that almost all NF-Y proteins have a negative average hydrophobicity index (GRAVY) value, except for GelNFYC4 (0.055), whereas the GRAVY values of the other proteins ranged from −0.939 (GmeNFY5) to −0.131 (GelNFY18). Therefore, most NF-Y proteins are hydrophilic, though their hydrophilicity varies in degree.

### Conserved motif and gene structure analysis of NF-Ys

**Figure 3:**
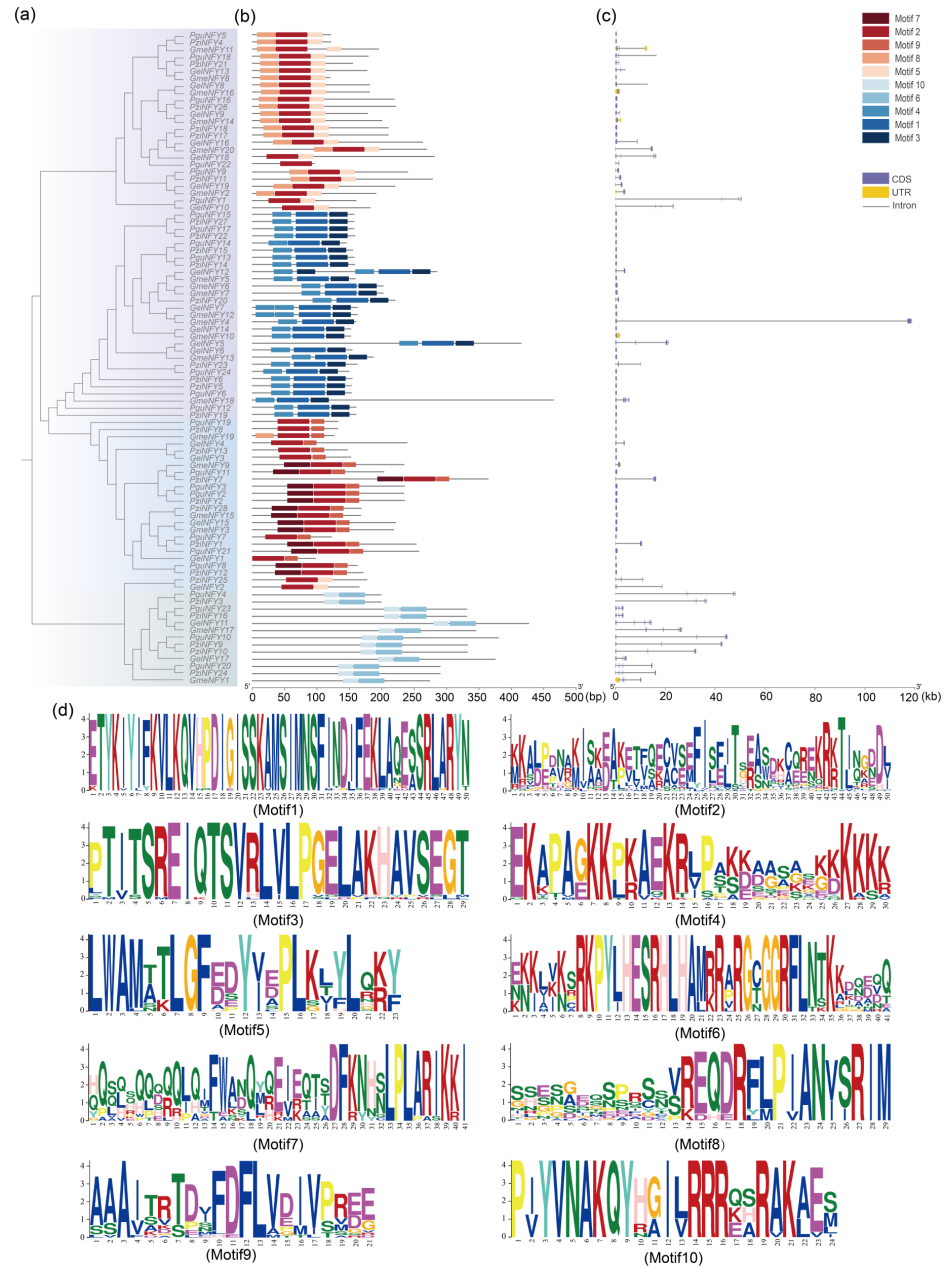
The phylogenetic tree, protein conserved motifs, and gene structures of the NF-Ys. (A) The maximum-likelihood (ML) phylogenetic tree of orchids NF-Ys. (B) The conserved motif patterns of NF-Y proteins. (C) The gene structures of *NF-Y* genes. (D) Logos of conserved motifs 1–10.

The conserved motifs of NF-Ys were further analyzed and visualized using the MEME website. The analysis identified a total of ten conserved motifs (Fig. 3). Motifs 6 and 10 were detected in all NF-YA subfamily members, motifs 1, 2, 3, 4, 5, and 8 were detected in all NF-YB subfamily members. In the NF-YC subfamily, almost all members contain motif 2, while a subset of proteins additionally harbor motifs 9 and 7; only a few members possess motif 5. In addition, analysis of NF-Y genes in orchids based on genome annotation (GFF files) revealed. A notable observation in the NF-YA subfamily is that members containing only motif 6 and motif 10 exhibit longer introns and more complex structures. Furthermore, coding sequences (CDS) and untranslated regions (UTRs) were distributed differently among these genes, and genes with longer introns also exhibited a higher number of CDSs. The complexity of these gene structures likely contributes to the functional diversity of NF-Y proteins.

### Chromosomal localization and collinearity analysis of orchid NF-Y genes

We visualized the distribution of *NF-Y* genes on chromosomes. The 24 *PguNFY*s are distributed on 15 chromosomes of *P. guangdongensis* (*PguNFY24* on an unknown chromosome), among which Chr05 has three *PguNFY*s, and the remaining chromosome contains one or two *PguNFY*s. The 28 *PziNFY*s are distributed on 16 *P. zijinensis* chromosomes, of which Chr03 contains four *PziNFY*s, and the remaining chromosome carrying one to three *PziNFY*s. A total of 19 *GelNFY*s are dispersed across 12 *G. elata* chromosomes, with each chromosome containing one or two *GelNFY*s. Specifically, Chr 05, Chr07, Chr08, and Chr16 each contain a single *GelNFY*. The 20 *GmeNFY*s are distributed on 10 chromosomes of *G. menghaiensis*, with GmeNFY20 located on an unknown chromosome. Chr03 contains four *GmeNFY*s, while the remaining chromosome contains one to three *GmeNFY*s.

**Figure 4:**
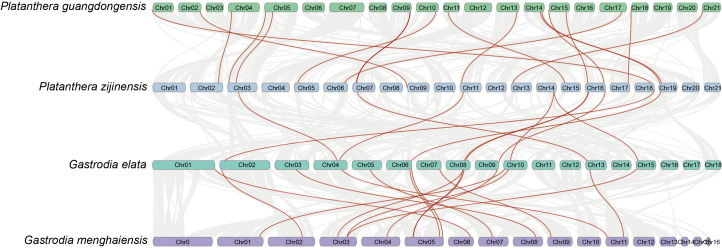
Collinearity analysis of orchids NF-Ys. The gray lines show collinear gene pairs between different species; the red lines show NF-Ys with collinear relationships between different species.

Furthermore, the collinearity between the *NF-Y*s of *P. guangdongensis*, *P. zijinensis*, *G. elata*, and *G. menghaiensis* was analyzed to investigate potential replication events in the evolution of the *NF-Y* genes in orchids. Despite the NF-Y genes being located at different positions across the genome, a one-to-one correspondence exists between NF-Y genes in different species, particularly within the same genus, such as *P. guangdongensis* with *P. zijinensis*, *G. elata* with *G. menghaiensis* (Fig. 4).

### *Cis*-elements prediction of NF-Ys promoter

**Figure 5:**
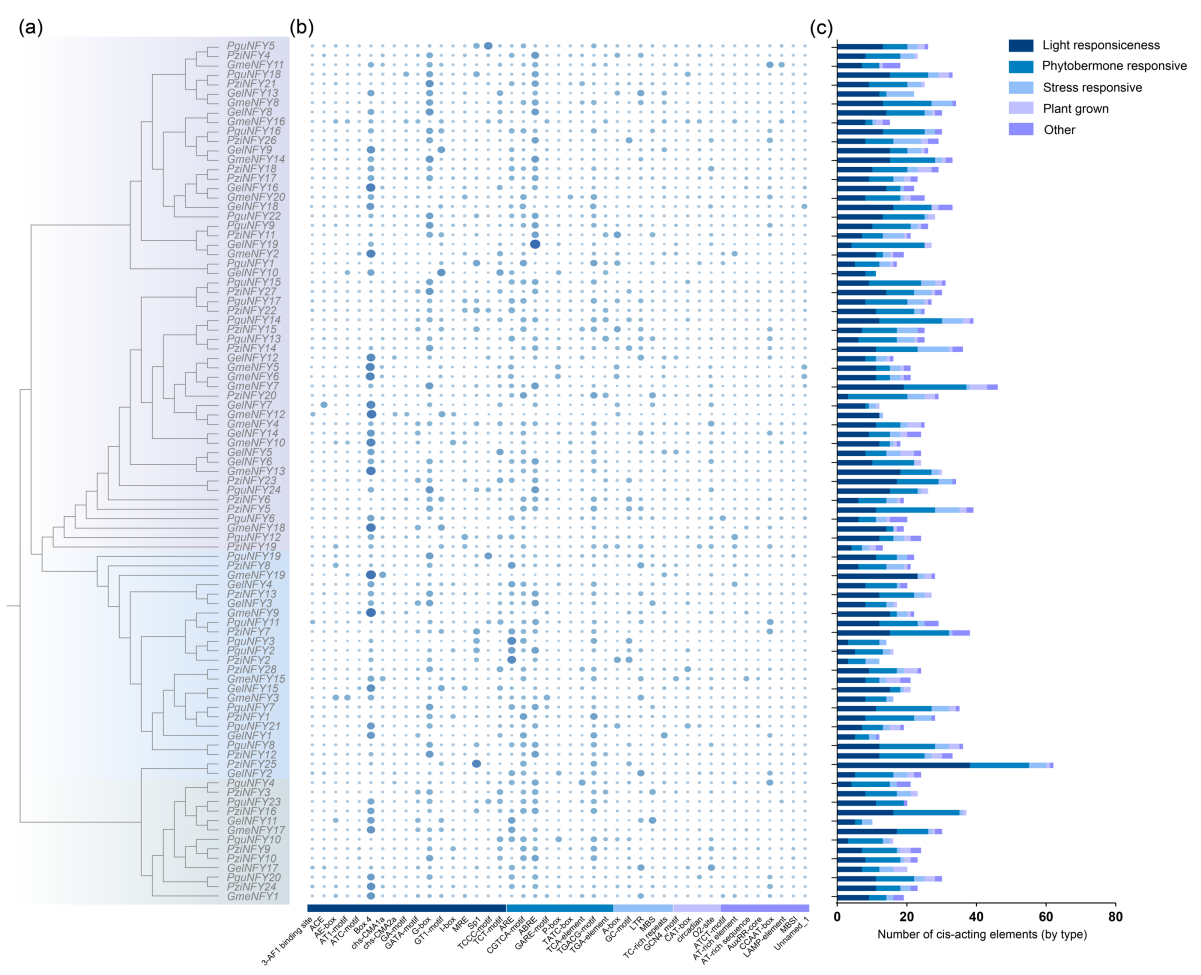
The *cis-*elements in the NF-Ys promoter region. (A) The phylogenetic tree of orchids NF-Ys. (B) the number of *cis*-elements in the NF-Ys promoter region; (C) the number of five broad categories. The types and quantities of *cis*-elements in Table S3.

Promoter elements were predicted within the 2,000 bp region upstream of the *NF-Y*s. Through *cis*-element analysis, a total of 2,253 *cis*-elements were identified, which were categorized into five categories: light responsiveness, phytohormone responsiveness, stress responsiveness, plant growth, and others. Light-responsive elements were the most abundant, with 42.23% (944/2,253) of the total (Fig. 5B). There are nine types of *cis*-elements related to plant hormone regulation, accounting for approximately 35.17% (786/2,253). In addition, five types of stress-related *cis*-elements and four types of plant growth *cis*-elements were identified, representing approximately 11.54% (258/2,253) and 5.68% (127/2,253) of the total, respectively (Fig. 5C, Table S3).

### Gene ontology analysis

**Figure 6:**
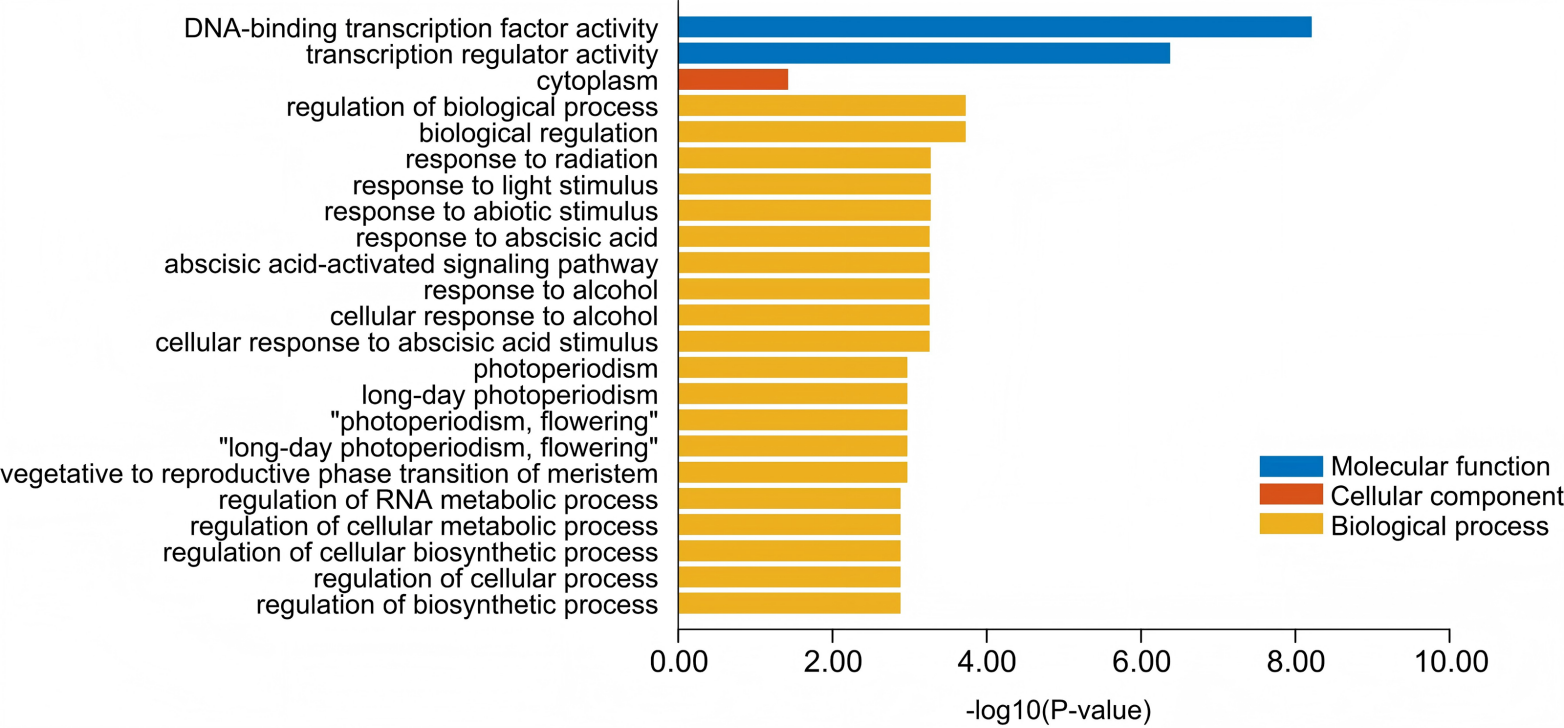
Gene ontology enrichment analysis of the identified NF-Y genes in four orchid species. The results of the GO annotation are provided in Table S4.

GO functional visualization based on eggNOG annotation of NF-Y genes in four orchid species revealed that these genes were significantly enriched in categories associated with plant growth, development, and floral organ formation (Fig. 6). At the molecular function level, genes were mainly enriched in DNA-binding transcription factor activity and transcription regulator activity, while enrichment in the cellular component category was relatively low. In contrast, substantial enrichment was observed in the biological process category, particularly in biological regulation and response to environmental stimuli. Notably, processes related to photoperiod and flowering, such as “photoperiodism” and “long-day photoperiodism, flowering” were also enriched, indicating that photoperiodic regulation plays a crucial role in orchid flowering and developmental processes, involving extensive gene participation and providing valuable clues for further functional studies of orchid genes (Table S4).

### Expression pattern and qRT-PCR analysis of GelNFYs

**Figure 7:**
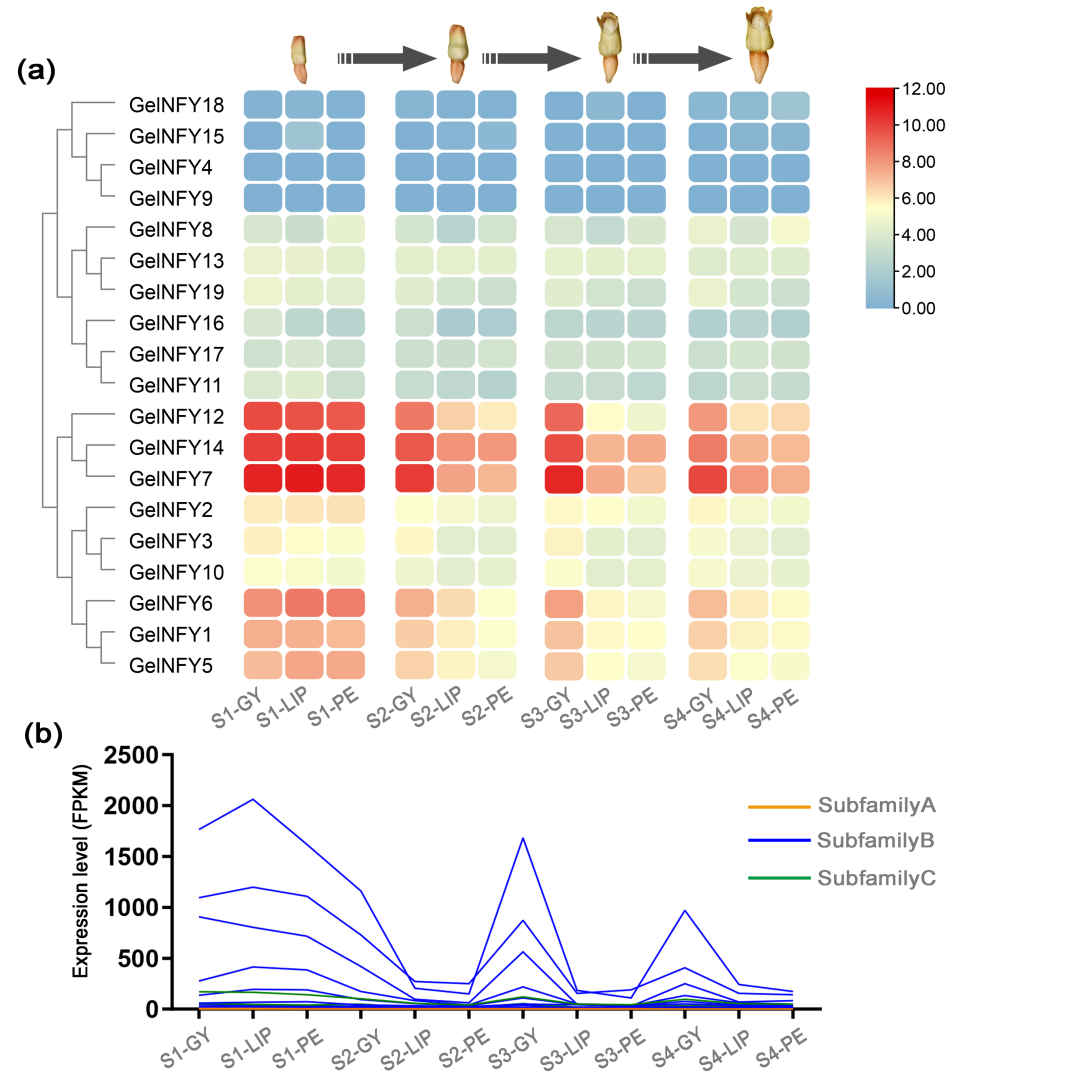
Expression pattern of GelNFYs in the floral component of *G. elata*. (A) Heatmap of the FPKM values of GelNFYs (Table S5). (B) A line chart depicting the expression levels of the three subfamilies at different times and different sites was the basis for screening the candidate genes of qRT-PCR.

The statistical power of this experimental design, calculated in RNASeqPower, was 0.78. Based on *G. elata* transcriptome data (Table S5), we analyzed the expression patterns of the GelNFYs in three flower organs: lip (LIP), gynostemium/ovary (GY), and perianth tube (PE) (Fig. 7). Most GelNFYs with the highest expression were observed in LIP1. In the NF-YB subfamily, GelNFY15 and GelNFY18 were almost unexpressed across floral organs, whereas GelNFY4 and GelNFY9, belonging to the NF-YC subfamily, also showed nearly undetectable expression in these tissues. In addition, certain genes also exhibited tissue- or time-specific expression, with higher expression levels observed in the GY. In addition, it was found that GelNFY2, GelNFY3, and GelNFY10 exhibited similar expression patterns, and GelNFY6, GelNFY1, and GelNFY5 also have similar expression patterns and higher expression levels, particularly in GY. Therefore, we have selected GelNFY2, GelNFY6, and GelNFY14 for the next qRT-PCR experiment.

**Figure 8:**
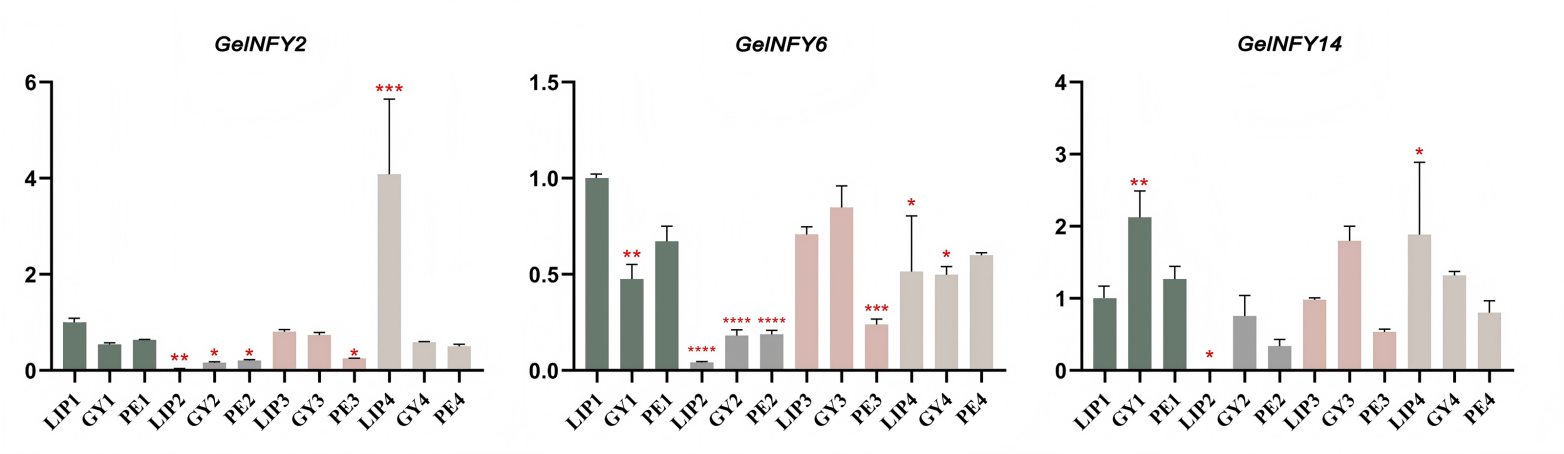
Real-time reverse transcription qRT-PCR verifying the effect of GelNFYs on flower organ development. The *Y*-axis represents relative expression values (2-ΔΔCT). Bars represent the mean values of three technical replicates ± SE (Table S7). The red asterisk indicates the *P* value in the significance test (* *p* < 0.05, ** *p* < 0.01, *** *p* < 0.001, **** *p* < 0.0001). Primers of GelNFYs are shown in Table S6.

The qRT-PCR results revealed that the expression trend of the selected genes was significantly correlated with the expression level of the transcriptome data (Fig. 8). In S2 (LIP2, GY2, and PE2), the expression levels of all three genes were markedly reduced, with the most pronounced decrease observed in LIP2. As floral development progressed, the three genes exhibited varying degrees of upregulation and were significantly upregulated in LIP4, suggesting their potential involvement in the regulation of lip development in *G. elata*.

## Discussion

The NF-Y transcription factor initially identified as a plant homolog of the CBF-B/HAP2 subunit in *Brassica napus* (Albani & Robert, 1995) has subsequently exhibited diverse responses to growth, development, and abiotic stress in various plants (Dai et al., 2021; Zhang et al., 2023). In this study, we identified 24 NF-Ys in *P. guangdongensis*, 28 NF-Ys in *P. zijinensis*, 19 NF-Ys in *G. elata*, and 20 NF-Ys in *G. menghaiensis*. The number of NF-Ys in these orchids was comparatively less than in most other plants, such as *A. thaliana* (36) (Kumimoto et al., 2010), *O. sativa* (34) (Niu et al., 2020), *Brassica campestris* (49) (Jiang et al., 2023b), *Phalaenopsi*s (24) (Jiang et al., 2023a), and *Malus domestica* (43) (Qu et al., 2020). In orchids, *G. elata* and *G. menghaiensis* harbor fewer NF-Y genes compared with photosynthetic orchids such as *Phalaenopsis* and *Platanthera*. This difference does not necessarily indicate a systematic contraction of the NF-Y family, but may instead reflect lineage-specific gene loss, differential retention following duplication events, or potential differences in genome assembly and annotation quality. Phylogenetic analysis revealed a close relationship among PguNFYs, PziNFYs, GelNFYs, GmeNFYs, AtNFYs, and OsNFYs, suggesting that *G. elata* may have lost some NF-Y genes during evolution to adapt to environmental changes (Fig. 2). A similar pattern has also been reported in other plant species, in which NF-YB genes generally outnumber those of the NF-YA and NF-YC subfamilies (such as *Nicotiana tabacum*) (Tian et al., 2024). The relatively high abundance of NF-YB genes in plants has been proposed to result from subfamily-specific expansion and higher retention rates following gene duplication events, possibly due to distinct functional constraints acting on NF-YB proteins. An analysis of the physicochemical properties of NF-Y proteins revealed that nearly all NF-Y proteins are hydrophilic. Most NF-Y proteins exhibited conserved physicochemical characteristics, consistent with previous studies on NF-Y in other plants (Petroni et al., 2012). Furthermore, many NF-Y proteins displayed II value exceeding 40, indicating their structural instability.

Gene replication events are integral to gene innovation and the expansion of gene families, playing a crucial role in enabling organisms to adapt to diverse and complex environmental conditions. This process contributes significantly to the evolutionary plasticity and versatility required for survival across a wide range of ecological niches (Cannon et al., 2004; Xie et al., 2023). Building on previous studies of duplicate gene identification (Magadum et al., 2013; Sharma et al., 2024), we further analyzed the fragments of predicted duplicate genes and tandem repeat events. In this study, the evolutionary relationship among NF-Ys in four orchids were examined through intergenic colinearity analysis. Despite the significant reduction in the number of NF-Y gene family members in *G. elata*, a clear phylogenetic relationship is evident among these genes. This suggests the presence of a conserved association and potential co-evolution between NF-Ys in different orchids (Fig. 4).

The conserved motifs and gene structures of NF-Y genes were generally consistent with those reported in previous research (Jiang et al., 2023b; Zanetti, Rípodas & Niebel, 2017), and there are significant differences in domains among different subfamilies. Comparative analysis of gene structure revealed that NF-YA subfamily genes had more exons (Fig. 3). Notably, multiple sequence alignments of amino acid sequences showed a high degree of conservation in the evolutionary process of the NF-Y gene family (Li et al., 2019; Madhu et al., 2024; Zhao et al., 2016). Consistent with studies on other plants such as *Phalaenopsis* (Jiang et al., 2023a) and *Petunia hybrida* (Li et al., 2019), the amino acid sequences of NF-Ys not only contain a DNA-binding domain but also subunit interaction regions (Fig. 3).

The dynamic network of *cis*-elements is essential for gene regulation. Promoter analysis revealed that the promoter region of the NF-Ys contains a significant number of elements associated with light and hormone responses (Box 4, ABRE), abscisic acid associated elements (CGTCA-motif, TGACG-motif, and ABRE), and plant growth-related elements (CAT-box and O2-site). Among these elements, Box 4 and ABRE are the most abundant (Fig. 5). Previous studies have demonstrated that NF-YCs regulate flowering time primarily through a photoperiodic pathway (Kumimoto et al., 2010). In *A. thaliana*, the NF-Ys in the light-signaling pathway promoted the transcription of SOC1, thereby facilitating flower development (Zhao et al., 2016). The involvement of NF-Ys in photoperiodic flowering response has been documented in various plants including *O. sativa* (Kim et al., 2016), *Populus tomentosa* (Li et al., 2022a), *Chrysanthemum indicum* (Wang et al., 2022) and others. Furthermore, abscisic acid plays a crucial role in many physiological processes and abiotic stress responses in plant growth (Emenecker & Strader, 2020). These findings suggest that NF-Ys may be involved in the response to light and plant hormones.

The regulatory function of a gene is directly influenced by its expression pattern. Although there are few studies specifically addressing the function of the NF-Y genes in orchids, transcriptome-level analyses have confirmed the presence and functionality of these genes across a wide range of plant species. NF-Ys of species such as *A. thaliana*, rice, peach, *Chrysanthemum*, and *Phalaenopsis* are expressed in various tissues, including roots, stems, flowers, and leaves (Zanetti, Rípodas & Niebel, 2017; Zhang et al., 2023). A proposed model suggests that the PhNFY complex, in response to low-temperature conditions, may regulate the expression of downstream genes involved in the floral transition, potentially triggering flowering in *Phalaenopsis* (Jiang et al., 2023a). While reports on the NF-Y gene in orchids are limited, transcriptome studies in other plants provide valuable insights into NF-Y function. For instance, the AtNF-YB gene in *A. thaliana* has been implicated in flowering regulation (Kumimoto et al., 2010). Overexpression of OsNF-YC6 in rice was verified to promote flowering under long-day (LD) conditions, suggesting that it can function as a flowering promoter (Kim et al., 2016). In peach, the genes PpNF-YB10 and PpNF-YC6 are specifically expressed in reproductive tissues, indicating their involvement in flower organs and fruit development (Li et al., 2022a). The expression of 18 BcNF-Ys was significantly upregulated in Chinese cabbage during the budding and rapid-bolting stages, suggesting that involvement in the development of this species (Jiang et al., 2023b). RNA interference (RNAi) of OsNF-YB1 expression leads to altered gene expression in cell cycle pathways, resulting in seed abnormalities with embryo and endosperm defects (Sun et al., 2014). TaNFYC11 is regulated by light and participates in the regulation of photosynthesis related gene expression (Stephenson et al., 2010), while overexpression HvNF-YB1 in barley accelerates flowering (Liang et al., 2012). Moreover, overexpression of TaNFYC11 in tomato, induced by SlymiR169c, significantly downregulates the accumulation of SLNF-YA1/3 and other gene transcripts, thereby enhancing drought resistance (Zhang et al., 2011). In this study, transcriptome data revealed that the level of NF-Ys regulation was different in different stages and organs. The expression levels of all GelNFY subfamilies peaked at S1, whereas the NF-YA subfamily exhibited little to no expression during subsequent floral organ development. From S1 to S4, expression changes in both the PE and GY were mainly associated with NF-YBs, with possible involvement of the NF-YC subfamily (Fig. 7). To further investigate the function of the NF-Ys in *G. elata*, we selected three GelNFYs with varying expression levels in different floral organs based on transcriptome data for qRT-PCR. The marked reduction in expression of GelNFY2, GelNFY6, and GelNFY14 at the S2 (LIP2, GY2, and PE2), particularly in LIP2, followed by significant upregulation in LIP4 during the blooming stage, suggests that these genes may play important roles in regulating lip development in *G. elata* (Fig. 8).

Collectively, the current findings support that the NF-Y gene family in Orchidaceae may play a role in regulating lip and gynostemium development and is involved in the flowering processes of *G. elata*. Our study of NF-Y gene expression in floral organs not only deepens our understanding of their involvement in flower development but also provides new perspectives for future research and molecular breeding efforts focused on orchid flower organ development.

## Conclusions

This study identified a total of 91 NF-Ys from four orchids: *P. guangdongensis* (24), *P. zijinensis* (28), *G. elata* (19), and *G. menghaiensis* (20). These NF-Ys were divided into three subfamilies according to phylogenetic relationships, with the NF-YB subfamily representing the largest proportion. NF-Ys within the same subfamily have the same conserved domains and similar gene structures, with the NF-YB subfamily displaying the most complex gene structure, suggesting it may have more significant regulatory functions. Most members of the NF-Y gene family have light-responsive regulatory *cis*-elements and functions related to floral organ development. Overall, GelNFY2, GelNFY6, and GelNFY14 exhibited coordinated downregulation at the S2 stage and significant upregulation in LIP4, highlighting their potential roles in the regulation of lip development in *G. elata*. Collectively, these findings provide a systematic overview of the NF-Y gene family in orchids with saprophytic-like characteristics, integrating gene family composition, structural features, and expression dynamics. This study extends current knowledge of NF-Y transcription factors in Orchidaceae and offers a comparative framework for exploring their potential roles in flowering regulation and developmental processes in orchids.

##  Supplemental Information

10.7717/peerj.21105/supp-1 Supplemental Information 1 The Protein Sequence of NF-Ys

10.7717/peerj.21105/supp-2 Supplemental Information 2 Characteristics of the NF-Y proteins from orchids

10.7717/peerj.21105/supp-3 Supplemental Information 3 Cis-elements of NF-Ys

10.7717/peerj.21105/supp-4 Supplemental Information 4 The Gene ontology analysis of NF-Ys

10.7717/peerj.21105/supp-5 Supplemental Information 5 The FPKM values of GelNFYs

10.7717/peerj.21105/supp-6 Supplemental Information 6 qRT-PCR primers

10.7717/peerj.21105/supp-7 Supplemental Information 7 The cq value of the qPCR

10.7717/peerj.21105/supp-8 Supplemental Information 8 MIQE Checklist

## Additional Information and Declarations

### Data Availability

The following information was supplied regarding data availability: The raw data are available in the Supplemental Files and at the China National GeneBank (CNGB): CNP0008391.
